# Changing patterns of family formation among internally displaced populations in Yemen: evidence from cross-sectional surveys

**DOI:** 10.1186/s12889-024-20889-9

**Published:** 2024-12-03

**Authors:** Shatha Elnakib, Linnea A. Zimmerman, Bothaina Attal, Tareq Alkebsi, Aisha Aldaram, Abdullah Al Kaff, Kate Mieth, Court Robinson

**Affiliations:** 1 Johns Hopkins Bloomberg School of Public Health, 615 N Wolfe St, Baltimore, MD 21231 USA; 2 Faculty of Medicine and Health Sciences, Sana’a University, Sana’a, Yemen; 3 Centre for Business Research, Cambridge Judge Business School, Cambridge, UK; 4 Central Statistical Organization, Sana’a, Yemen; 5 Building Foundation for Development, Sana’a, Yemen

**Keywords:** Child marriage, Early childbearing, Conflict, Humanitarian settings, Family formation, Adolescent health, Middle East, Sexual and reproductive health

## Abstract

**Introduction:**

: Yemen has one of the highest rates of child marriage in the Middle East and North Africa region and is home to one of the world’s worst humanitarian crises. How the conflict and resulting displacement have impacted family formation patterns is not entirely clear. In this study, we investigate the impact of displacement on child marriage and early childbearing by comparing time-to-first-marriage and time-to-first-birth among displaced and non-displaced girls.

**Methods:**

We used data collected through cross-sectional multistage stratified cluster household surveys in three governorates in Yemen with high concentrations of internally displaced persons (IDPs). Employing an analytic sample 1,861 girls aged 15–24, we compared entry into first marriage and time to first birth between IDPs and non-displaced persons using Kaplan Meier curves and Cox regressions.

**Results:**

We found high rates of child marriage in this population, with 37.8% and 34.5% of ever-displaced and never-displaced girls aged 20–24 married before age 18. Overall, those who were displaced had 30% higher hazard of overall marriage compared to their host counterparts (95% CI 1.16–1.47), and 23% increased hazard of child marriage (95%CI 1.01–1.51). Stratification by governorate revealed heterogeneity across governorates, with displaced populations at higher hazard of marriage in Aden and Maarib but not in Hadramout. We found that child brides and displaced participants were more likely to initiate earlier childbearing compared to non-child brides (HR = 3.44; 95% CI 2.94 4.0) and host counterparts (HR 1.35; 95% CI 1.15–1.58). Despite generally having lower age at first birth, child brides experienced a 19% lower hazard of first birth after marriage compared to those married over 18, indicating that child brides tended to wait longer before first birth (95% CI 0.72–0.92). We did not find a significant association between displacement status and marriage to birth interval after adjusting for child marriage status.

**Conclusion:**

Our results highlight the impact of forced displacement on time-to-first-marriage and suggest that those who are displaced are more vulnerable to child marriage. We did not find compelling evidence for child marriage driving an increase in early childbearing. Nonetheless, our findings highlight the need for interventions that focus on prevention and mitigation of child marriage in this setting, particularly among IDPs who are at increased vulnerability.

## Introduction

Child marriage, defined as marriage or informal union under the age of 18, is a human rights violation. and is associated with adverse health and social impacts among girls and their offspring. Girls who marry early tend to have earlier and frequent pregnancies, resulting in higher-than-average maternal morbidity and mortality [1]. The practice is also associated with diminished agency and autonomy, increased risk of gender-based violence, and disruption of schooling and labor force participation [1–4]. Given both the implications for health and rights, efforts to eliminate child marriage have taken on increased urgency in the last two decades [1].

Of 650 million child brides alive today, 40 million, or 6%, reside in the Middle East and North Africa region, with the highest numbers in Sudan and Yemen [5]. The region’s remarkable progress in reducing child marriage rates has stagnated over the past decade, and recent fragility and conflict have increased concern that child marriage rates are rising and will continue to rise in the region [6]. In the Middle East, poverty, entrenched social norms that devalue girls’ education and labor force participation, and other harmful practices drive the practice [5]. Armed conflict, which has beset many countries in the region, is also speculated to have increased child marriage rates [5]. Yet a recent multi-country study indicates that the impact of conflict on child marriage is multi-faceted and context-specific [6]. In six humanitarian settings across the Middle East and South Asia, the authors found evidence in only one country for an association between conflict-induced displacement and child marriage. A 2015 systematic review of the impact of conflict on adolescent transitions, which mostly featured retrospective analyses of nuptiality and fertility rates, similarly found mixed evidence [7]. In some settings an increase in marriage rates or acceleration of marriage timing occurred during conflict, while in others, skewed sex ratios stemming from male-specific mortality, migration and military drafting caused a reduction in marriages, including child marriages [7].

In Yemen, child marriage is prevalent, with the country having one of the highest rates of child marriage in the Arab world. A survey conducted in 2006 by the government in collaboration with UNICEF found that 52% of girls were married under age 18, and 14% under the age of 15 [8] There is burgeoning evidence that the war has been associated with an increase in child marriage rates [6, 9, 10]. A study conducted by Elnakib et al. looking at child marriage in six humanitarian settings found that Yemen was the only country showing evidence of increase in child marriage [6]. Research by Hunersen et al. additionally found evidence for child marriage happening among boys but that girls were over four times more likely to experience child marriage compared to boys [9]. Qualitative data revealed that displacement influenced economic security and drove shifts in household power dynamics, which in turn affected decisions about marriage and limited the ability of girls to advocate for themselves [9].

The war which started in 2014 has resulted in an acute humanitarian crisis, with more than half the population found to be acutely food insecure and over 4.5 million internally displaced [11]. Violent insecurity, a worsening economic crisis, recurring infectious disease outbreaks and high levels of malnutrition have resulted in the collapse of the country’s health system. Across the country, almost half of all health facilities are out of service or only partially functioning due to staff shortages, infrastructural damage, and unavailability of fuel [11]. The ongoing conflict is the result of long-standing political, economic, and social tensions between the north and south of the country [9]. In late 2014, the Houthi movement captured Sana’a, the capital, and in reaction, a Saudi-led coalition of Arab states with US and UK backing launched a campaign to reinstate the government [12]. Fighting between both parties has triggered substantial displacement and destruction, and deliberate and indiscriminate attacks against humanitarian staff, combined with restrictions on humanitarian access, have interrupted access of Yemenis to life saving humanitarian assistance. While large-scale fighting has not resumed since 2022 due to a truce facilitated by UN-mediated negotiations, displacement continues to occur with nearly 276,000 Yemenis displaced in 2022 [12]. Displaced populations have to wrestle with persistent insecurity and heightened livelihood insecurity.

Before the conflict, child marriage was widely practiced in the country. The most recent Demographic and Health Survey, conducted in the country in 2013, revealed that 32% of Yemeni girls married prior to age 18 and one in ten married before 15 [13]. The Yemeni Personal Status law does not establish a minimum age of marriage and attempts to introduce a law that sets the age at marriage at 15 have been met with staunch resistance. The eruption of conflict and the ensuing humanitarian crisis have positioned child marriage as a coping mechanism and a means to protect girls and relieve families of the cost of caring for them [9].

The influence of humanitarian emergencies on adolescent fertility following marriage is less well established. There is substantial evidence supporting the association between child marriage and higher lifetime fertility, as child marriage prolongs women’s sexual exposure risk, increasing exposure to pregnancies over the reproductive life span [14]. Similarly, child marriage is associated with younger age at first birth, again due to earlier sexual debut [14, 15]. Adolescent childbearing, particularly before age 18, is associated with increased risk for a range of poor maternal and newborn health outcomes, including death, relative to women age 20–24 [16, 17] and risks for poor outcomes may be exacerbated during humanitarian emergencies when health systems are weakened. Efforts to promote delayed childbearing among adolescents in emergencies may be a means to reduce high risk pregnancies and lower maternal mortality, but there is little evidence on whether humanitarian emergencies independently influence time to childbearing following marriage. Evidence has shown heterogenous effects on fertility following humanitarian emergencies [18], but no papers have specifically explored how displacement during an emergency influences the transition from marriage to parenthood.

How humanitarian settings impact on marriage timing and the extent to which child marriage is followed closely by childbearing in these settings are important considerations for humanitarian actors trying to improve adolescent sexual and reproductive health outcomes. This paper set out to investigate how the unfolding humanitarian crisis in Yemen impacts marriage timing, child marriage incidence, and fertility behavior in three governorates with high concentrations of internally displaced populations.

## Methods

### Overview of study design

**Fig. 1:**
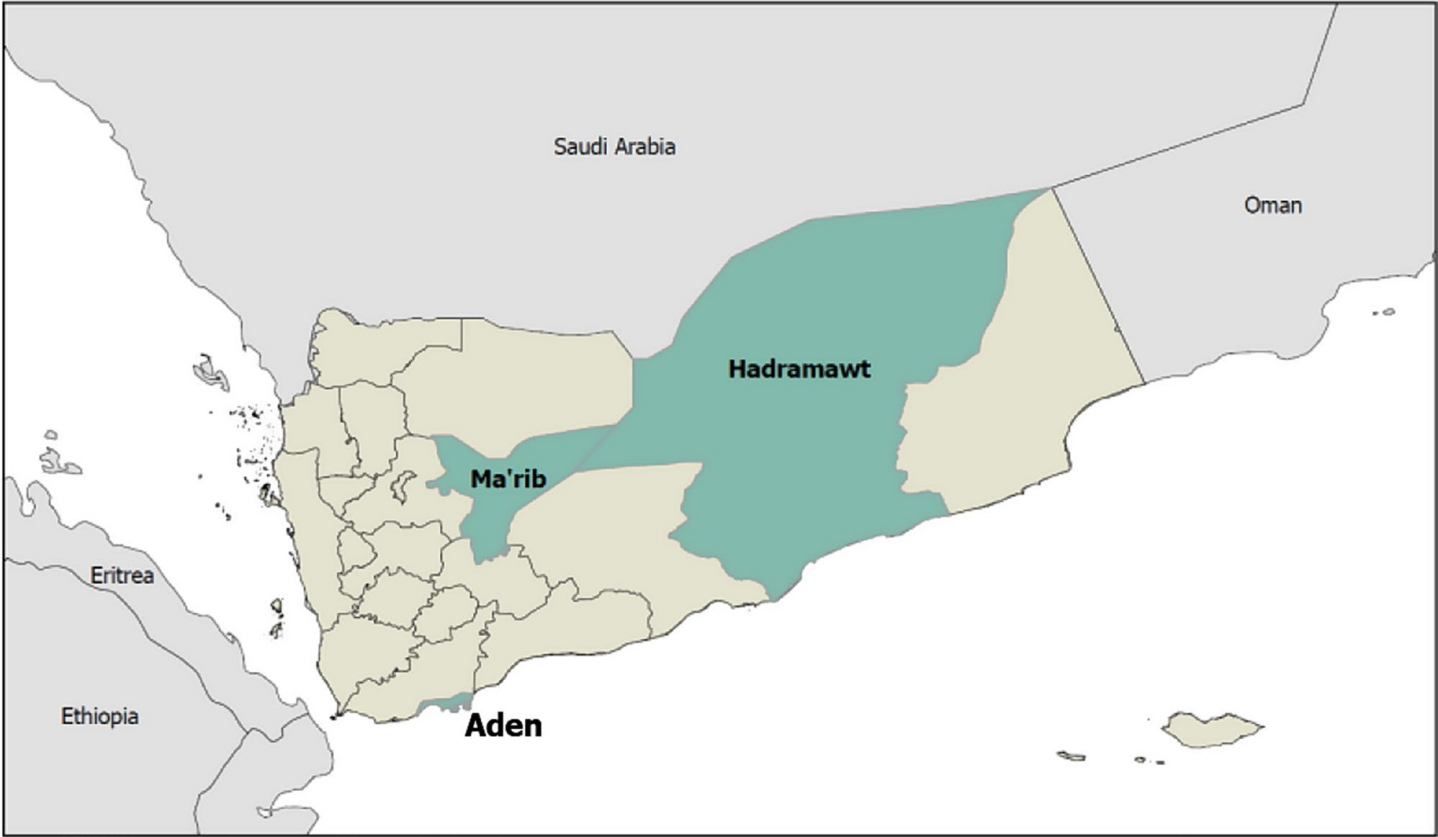
Map of study sites

A total sample size of 1600 households was calculated with the goal of ensuring precision of prevalence estimates. Assuming a prevalence of child marriage of 50% and 95% confidence, a sample size of 385 was needed to obtain estimates with 5% margin of error in each of the three strata. This number was inflated to 800 to account for an assumed design effect of 2. To obtain this number of girls, we selected 1600 households and interviewed up to two girls in each household.

The 1600 households were disproportionately allocated across the three governorates. Given that Ma’arib has a large number of IDPs, we oversampled participants there. In total, we selected 400 IDPs and hosts in Aden, 400 IDPs and hosts in Hadramaut, and 800 IDPs and hosts in Ma’arib. Hosts were defined as those living in close proximity to the IDPs included in the study.

### Data collection

The data used for this study come from the R2HC funded Early Marriage Early Childbearing study, a collaborative study between Building Foundation for Development, University of Sana’a, Johns Hopkins Bloomberg School of Public Health (JHSPH). Quantitative and qualitative data were collected between and November and December 2022 in three governorates in Yemen.

All data collection was carried out face-to-face by local Arabic-speaking data collectors, who were trained in human subjects research and the study protocol. Data collection took on tablets programmed with Kobo Toolbox.

### Eligibility criteria

Households were eligible for participation in the survey if there was at least one female age 15–24 that had been living in the household for at least one month in the previous 12 months. Interviewers screened the household for eligibility by asking the eldest female household resident if there were any married or unmarried females aged 15–24 who had lived in the household for at least one month in the previous 12 months. If not, the household was not eligible, and the interviewer proceeded to the next nearest household. If there was at least one married female age 15–24, then the interviewer asked the respondent if the household moved to the current location since October 2018. If yes, the interviewer then asked if the move was due to conflict. If the household moved since 2018 but did not move due to conflict, then the household was eligible to participate and was defined as host. If the household had been a resident since before November 2018 (defined as host) OR if they moved since October 2018 (defined as IDP) due to conflict, the household was eligible. Once eligibility was confirmed, the adult was consented orally and administered the household questionnaire. Subsequently, the eligible adolescent was consented or assented in case the respondent was an unmarried female under age 18 and parental permission was sought. Up to two randomly selected adolescents were interviewed within each household.

#### Dependent variables

*Time to first marriage (in years). Female respondents aged 15–24 were asked if they were married*,* and if so*,* the age at which they married.*

*Time to first birth (in years): Married female respondents 15–24 were asked if they were married*,* and if so*,* the age at which they married.*

Interval between marriage and first birth: Married respondents were then asked if they have given birth and if so, the age at which they gave birth for the first time. We calculated the interval between marriage and first birth by subtracting age at first birth from age at first marriage.

Since we investigate both marriage and first birth, we use two different analytical samples and dependent variables.

#### Independent variables

For first marriage, our primary explanatory variable is displacement status, categorized as host vs. IDP. For first birth, our explanatory variable is displacement status as well as child bride status (defined as whether the respondent was married before age 18 or not).

#### Analytic sample

For analyses exploring first marriage, our analysis included all adolescents aged 15–24 (*n* = 1,879). We excluded any adolescents who had missing information on age (*n* = 18) This resulted in a final analytic sample of 1,861 adolescent girls.

For analyses exploring first birth, in time-to-event models, we included only ever-married adolescent females (*n* = 1,057) and excluded those whose pregnancies occurred prior to their marriage since extramarital pregnancy is rare in this population. This resulted in a final analytic sample of 1,043 females for time-to-event analyses.

### Analytic models

All analyses were stratified by displacement status. We first generated frequency estimates of marriage and childbearing by age and standard child marriage indicator measures. To account for right censoring, subsequent analysis used survival methods. Kaplan-Meier estimates of marriage-free probabilities were calculated and were compared using the log rank test between host and displaced girls. Cox Proportional hazards models were used to estimate hazard ratios of child marriage and their confidence intervals. Survival time was defined as the age of respondent at first marriage, while for those who were unmarried at the time of the survey, survival time was defined as their age at the time of the survey. A censoring variable was created such that girls who were either not married at the time of the survey, or who reached age 25 without getting married were censored. The proportional hazards assumption was assessed using Schoenfeld residuals for each independent variable. Our analysis did not violate the proportional hazards assumption and so we presented findings for Kaplan Meier and Cox Proportional Hazard models. We then estimated the hazard ratios of marriage prior to age 18, comparing hosts to displaced populations.

In the second set of analyses concerning time to first birth, we set the origin at marriage and we tested differences in time to first birth, measured in years, by displacement status and by age at marriage, comparing women who were married before age 18 to those married after age 18. Observations were censored at exact year since marriage, first birth, or at three years post marriage due to sample size issues.

### Ethics approval

This study was approved by the Institutional Review Board of the Johns Hopkins Bloomberg School of Public Health (reference number: 21168). The data used contained no personal identifying information of any study participants, and all participants provided verbal consent/assent. Local IRB approval was obtained from the Ethics Review Committee of the Ministry of Health and Population in Aden (reference number: GAIR/004). We obtained verbal Informed consent from adult participants and married girls ages 16–17 who are considered emancipated minors in this context. Oral assent coupled with permission from a parent/guardian was obtained from unmarried children 10–17 and married children under age 16.

## Results

A total of 1,879 married and unmarried adolescent girls aged 15–24 were included in our sample. Table 1 below summarizes the demographic characteristics of the sample. Roughly half were in the age cohort 15–19 (52%) and the other half was in the 20–24 age cohort (48%). A total of 1057 women were married, divorced, or widowed, constituting 57% of the sample. Of those, 1861 had information on age of marriage and were included in the child marriage analysis.

**Table 1:** Sociodemographic characteristics of respondents

	Total Sample *N* = 1,879		Ever displaced *N* = 966		Never displaced *N* = 913	
	N	%	N	%	N	%
*Total Sample*						
Age						
15–19	987	52.5	521	46.1	466	51.0
20–24	892	47.5	445	53.9	447	49.0
Mean age	19.3	Sd = 3.0	19.1	2.9	19.5	3.0
Marriage status						
Married	996	53.5	557	58.3	439	48.5
Widowed	19	1.0	14	1.5	5	0.6
Divorced	32	1.7	14	1.5	18	2.0
Separated	10	0.5	5	0.5	5	0.6
Engaged	133	7.2	65	6.8	68	40.9
Single	671	36.1	301	31.5	370	7.5
Currently in school						
Yes	567	65.1	253	31.5	314	61.7
No	1,057	34.9	551	68.5	506	38.3
Perceived SES status (natal family)						
Very rich	4	0.2	0	0.00	4	0.44
Rich	101	5.4	19	2.0	82	9.0
Comfortable	1,042	55.5	462	47.8	580	63.5
Poor	732	39.0	485	50.2	247	27.1
Ever-married (*N* = 1,057)						
Age at first marriage						
< 15	105	10.0	64	10.9	41	8.8
15–17	474	45.0	270	46.0	204	43.9
18+	473	45.0	253	43.1	220	47.3
Number of times married						
1	1,037	98.1	583	98.8	454	97.2
2	19	1.8	7	1.2	12	2.6
3	1	0.1	0	0	1	0.2
Place of marriage among displaced girls						
Gov of origin			262	44.4		
Gov where survey is being done			301	51.02		
Other gov (3rd gov)			27	4.6		
Timing of marriage among displaced girls						
Married before displacement			245	25.4		
Married after displacement			701	72.6		
Married at the same time of displacement			20	2.1		
Parity						
Nulliparous	350	33.1	202	34.2	148	31.7
Parous	707	66.9	388	65.8	319	68.3

### Child marriage

Table 2 presents information on standard child marriage indicators. Overall, 26% of adolescent girls aged 15–19 were married under age 18, and 33% of them were currently married. Among the older age cohort – girls 20–24 – child marriage prevalence was 36%. While it seems like child marriage rates were higher among the older age cohort, the younger cohort had not completed time at risk, and thus a better comparison can be made with the age cohort of girls aged 18–19. In this group, a comparable percentage of girls (36%) entered into marriage or union before age 18. Table 2 also compares child marriage indicators between those who reported ever being displaced and those who were never displaced. Those who were ever displaced had higher rates of marriage and child marriage across all age cohorts.

**Table 2:** Standard child marriage indicators

Indicator	Total		Ever displaced		Never displaced	
	N	%	N	%	N	%
Girls 15–19 currently married	316	32.5	200	38.4	116	24.9
Girls aged 15–19 married under 18	257	26.0	166	31.9	91	19.5
Girls 20–24 married under 18	322	36.1	168	37.8	154	34.5
Girls 18–19 married under 18	130	35.6	76	40.9	54	30.2

**Fig. 2:**
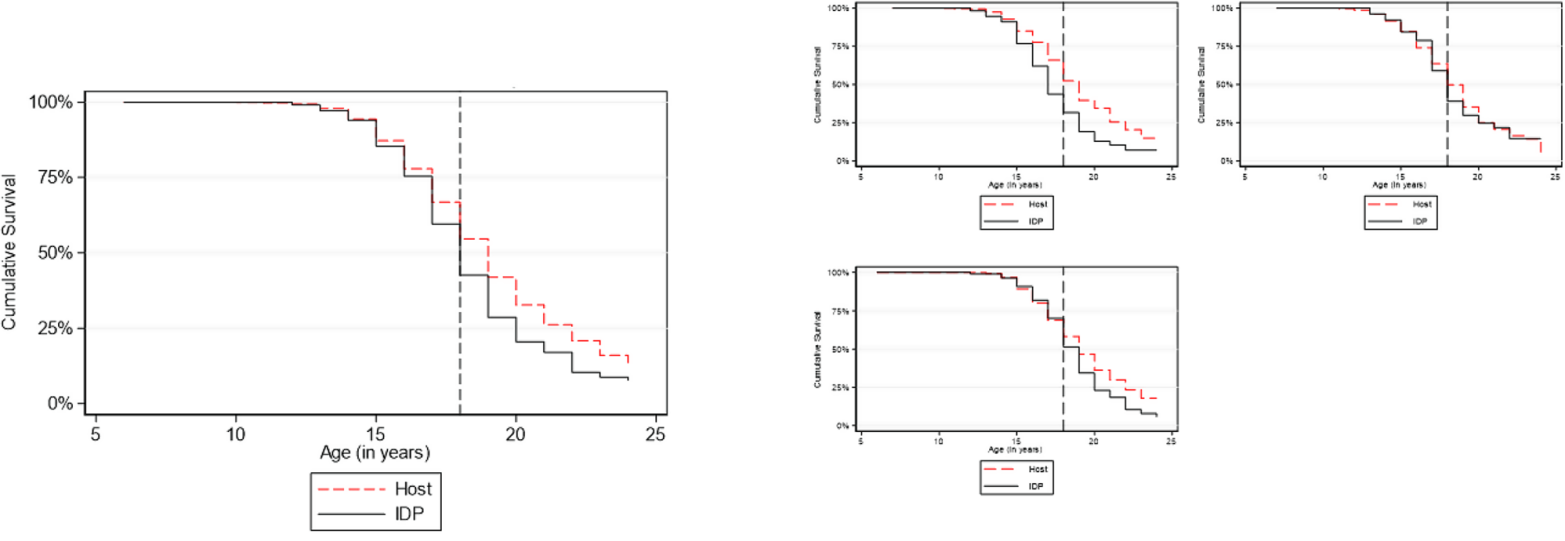
Survival function for age at marriage comparing hosts to displaced populations across and within the three governorates

Overall, a little less than 50% of the sample was married by age 18 (49.3%; 95% CI 46.7% − 52.0%), and by age 24, almost 90% girls were married. Figure 2 further demonstrates the survival distribution comparing those who are displaced, thereafter referred to as IDPs, to those not displaced, referred to as host populations, overall, and in each of the three governorates.

Table 3 shows the hazard of marriage, censored at age 25 and at age 18, and the incidence rate ratio of child marriage, comparing hosts to IDP populations across and within each of the three study governorates. Overall, IDP girls had 30% higher hazard of overall marriage compared to their host counterparts (95% CI 1.2–1.5), and 23% increased hazard of child marriage (95%CI 1.0–1.5). Stratification by governorate reveals that cumulative survival probabilities are heterogeneous across governorates, which is further confirmed by adding an interaction term in the Cox Hazard regression model (not shown). Hazard of marriage comparing displaced populations to hosts is higher in Aden and Maarib but not in Hadramout, and hazard of child marriage is only significantly higher among displaced populations in Aden.

**Table 3:** Hazard of marriage and child marriage comparing IDP to host populations among girls aged 15–24

	Total		Aden		Hadramaut		Ma’arib	
	Estimate	95% CI	Estimate	95% CI	Estimate	95% CI	Estimate	95% CI
Hazard ratio of marriage	1.3*	1.2–1.5	1.7*	1.3–2.2	1.05	0.8–1.3	1.3*	1.1–1.5
Hazard ratio of child marriage	1.2*	1.0–1.5	1.8*	1.3 – 2.62	1.09	0.7–1.6	0.9	0.7–1.3

*Significant at alpha = 0.05

### Early childbearing

**Figs. 3:**
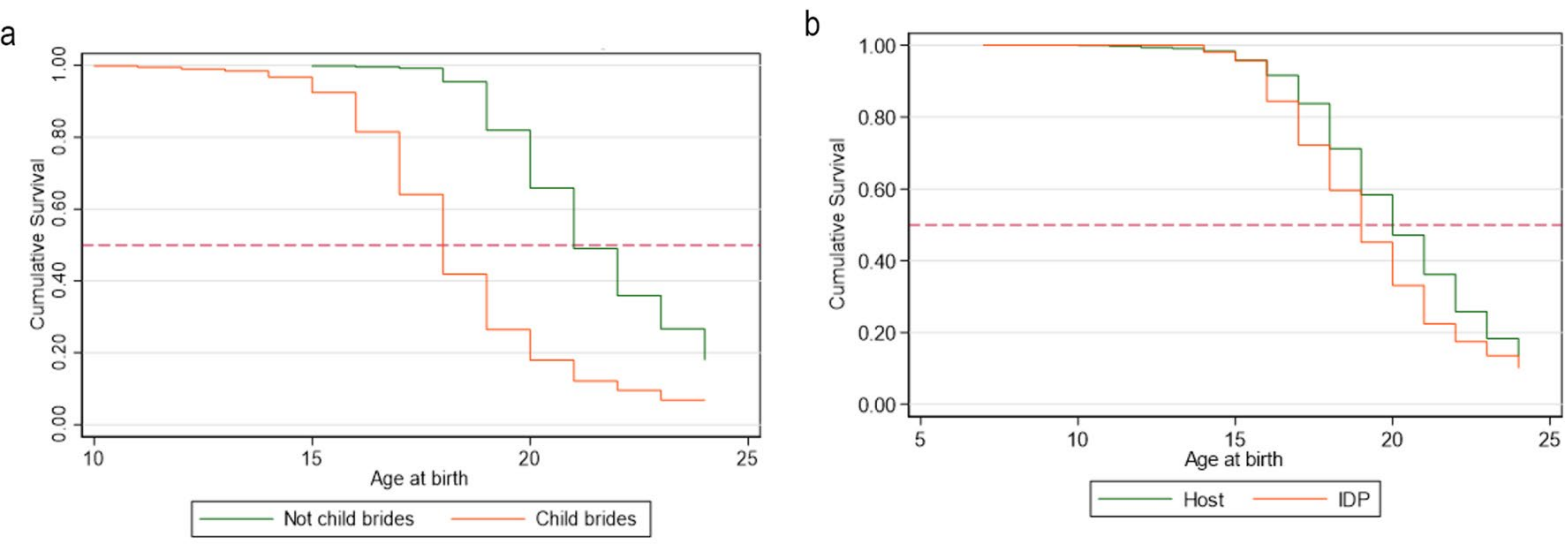
**a** and **b** Survival function for time to first birth (age at first childbearing) comparing girls married < 18 to those married $\:\ge\:$18 and comparing hosts to IDPs

In the overall sample, by age 15, 4.3% of the sample had initiated childbearing (95% CI 3.2% − 5.6%) and by age 18, 32.2% of the sample had their first child (95% CI 29.4% − 35.2%). Figure 3a and b compare age at first birth between child brides and non-child brides as well as IDPS and host populations. We find a significant difference in time to first birth between child brides and non-child brides with child brides having 3.4 times the hazard of first birth compared to their non-child bride counterparts (95% CI 2.9–4.0) (Table 4). Adjusting for displacement status, child brides are still more likely to initiate childbearing compared to girls married over the age of 18. Similarly, IDPs were more likely to initiate childbearing earlier than their host counterparts (HR 1.4; 95% CI 1.2–1.6). Adjusting for child bride status, displacement is still associated with earlier childbearing (Table 4).

**Table 4:** Hazard of first birth among girls married < 18 to those married $\:\ge\:$18 and comparing hosts to IDPs

	Total	
	Estimate	95% CI
Hazard ratio of birth (Ref: girls married $\:\ge\:$18)	3.4*	2.9–4.0
Hazard ratio of birth (Ref: hosts)	1.4*	1.2–1.6
Adjusted hazard ratio of birth (Ref: girls married $\:\ge\:$18)	3.4*	2.9–4.0
Adjusted hazard ratio of birth (Ref: hosts)	1.3*	1.1–1.4

*Significant at alpha = 0.05

**Fig. 4:**
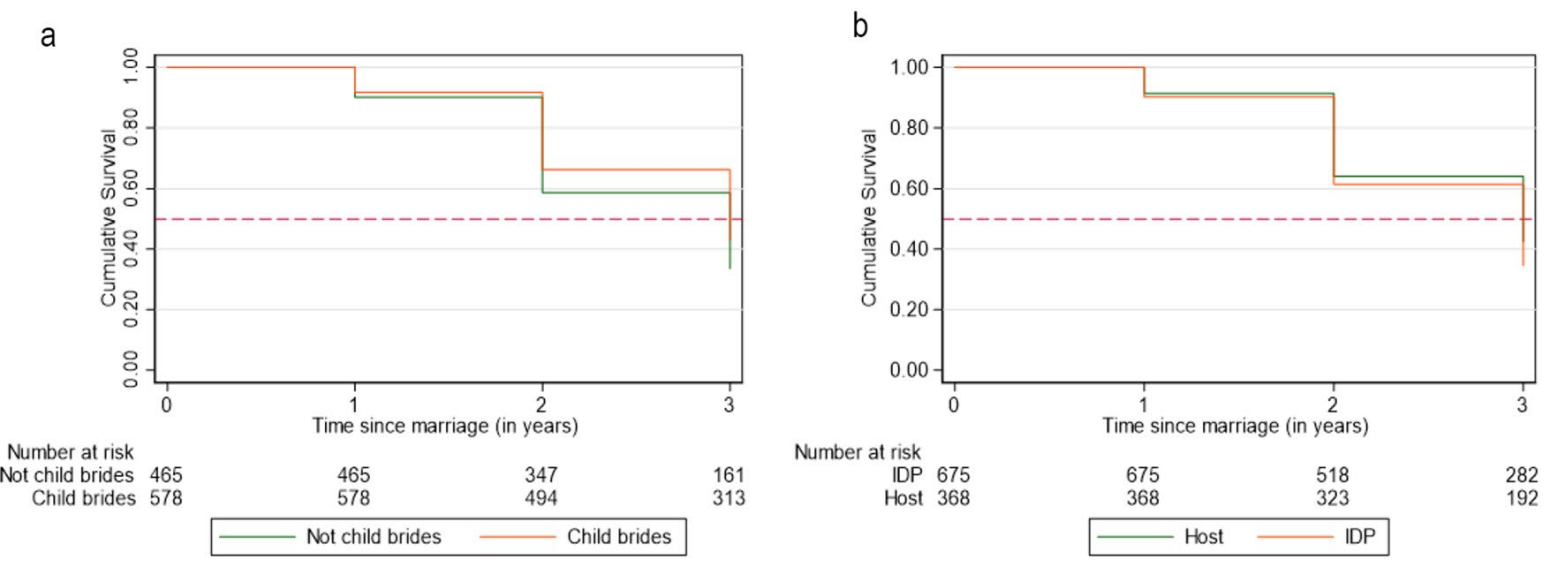
**a** and **b** Survival function for the interval between marriage and first birth comparing girls married < 18 to those married $\:\ge\:$18 and comparing hosts to IDPs

In the overall sample, 10% of participants had their first birth by year 1 of marriage (95% CI 7.4% − 10.9%), and by year 2, this estimate increased to 37% of participants (95% CI 33.9% − 40.0%). Figure 4a and b present survival curves for time to first birth following first marriage. We find a significant difference in cumulative probabilities of childbearing between the two groups, with child brides experiencing a 19% lower hazard of first birth in the first three years of marriage (HR 95% CI 0.7–0.9) (Table 5). The hazard of first birth in the first three years of marriage however was higher among IDPs compared to hosts, with IDPs experiencing 15% higher hazard (95%CI 1.0–1.3) (Fig. 4b). Even after accounting for displacement status, child brides continue to be at lower hazard of first birth in the first three years of marriage compared to those who are not child brides (aHR 0.8; 95% CI 0.7–0.9).

**Table 5:** Marriage to birth interval comparing girls married < 18 to those married $\:\ge\:$18 and comparing hosts to IDPs

	Total	
	Estimate	95% CI
Hazard ratio of birth in the three years after marriage (Ref: girls married $\:\ge\:$18)	0.8*	0.7–0.9
Hazard ratio of birth in the three years after marriage (Ref: hosts)	1.2*	1.0–1.3
Adjusted hazard ratio of birth in the three years after marriage (Ref: girls married $\:\ge\:$18)	0.8*	0.7–0.9
Adjusted hazard ratio of birth in the three years after marriage (Ref: hosts)	1.1	0.98–1.3

*Significant at alpha = 0.05

## Discussion

The question of how armed conflict and displacement affect adolescent transitions and family formation has important implications for humanitarian programming and response. Whether conflict and displacement expedite entry into marriage, and how that in turn affects timing and frequency of childbearing can help humanitarian actors determine and prioritize interventions to meet the needs of adolescents and young people. This study finds that IDPs in Yemen were more likely to enter into marriage earlier than their host counterparts. While the relationship between displacement status and marriage timing did not hold in Hadramout, it was the case in both Aden and Marib governorates, where timing of marriage occurred earlier among IDPs compared to hosts.

Why this is the case can potentially be explained by the nature of displacement in Aden as well as the governorates from which displaced populations originated. Aden is the capital city in the South and IDPs live alongside hosts, embedded in predominantly urban locations. Protection concerns in urban locations in Aden may be operating as drivers of child marriage in these settings. This aligns with evidence from other contexts including Egypt where protection concerns arising from residence in urban areas were cited as a reason to marry off girls at an early age [19]. Research by Women’s Refugee Commission finds that displaced populations residing in urban areas often report disconnection and alienation in their communities, erosion of social networks and sense of community in addition to a multitude of other risks associated with urban poverty [20]. Another potential explanation for the increased risk of child marriage among IDPs compared to hosts can be explained by difference in traditions around child marriage that vary across governorates. For example, over 80% of IDP respondents in Aden were displaced from Al Hudayda governorate where child marriage rates are higher than Aden and, according to the most recent Yemen Multiple Indicator Cluster Survey (MICS) conducted in 2022, 30.7% of women of reproductive age (WRA) in Al Hudayda were married under age 18 compared to 23% of those residing in Aden [21]. In contrast, child marriage rates are higher than the national average in Marib, with 35.8% of WRA married under 18, thereby potentially masking spikes in child marriage when comparing IDPs there to hosts [21].

That said, in each of the three governorates child marriage rates among IDPs were independently high, highlighting the need for cross-sectoral programming aimed at both prevention of child marriage and mitigation of its harmful impacts. Interventions that provide girls and their families with alternatives to marriage – through encouraging school attendance and retention as well as investing in economic opportunities for girls and women – have been shown to be effective approaches in stable settings [1, 22]. While the evidence on interventions addressing child marriage in humanitarian settings is more limited, the protracted nature of the humanitarian situation in Yemen makes it likely that such interventions may be as effective in this context. These findings point to the importance of investments in girls’ education and employment, as a means to alleviate pressures that drive child marriage in this context.

We also find that child brides and IDPs initiate childbearing earlier than their non-child bride and host counterparts. This is consistent with the assumption that early entry into marriage results in early initiation of childbearing and underscores the urgency to delay age at marriage in this setting. It also indicates that displacement and associated vulnerabilities may result in girls initiating childbearing earlier. Yet, while overall age of first birth tended to be lower for child brides, we find that those who are married as children tend to delay childbearing in the first three years of marriage compared to those who are married over the age of 18. This was the case irrespective of displacement status. Indeed, among both IDPs and hosts, there was a tendency to delay childbearing in the first three years post marriage among those who were married as children. The finding that childbearing does not necessarily occur immediately after marriage in this group is at odds with evidence from other settings which supports the link between child marriage and immediate childbearing [1, 23].

Whether these findings are unique to the Yemeni context or whether it is a pattern to be expected in other conflict settings is not entirely clear. Further research is needed to add to the evidence based on family formation and how it is impacted by conflict and displacement. Research investigating the impact of conflict exposure on fertility behaviors in other settings is limited and mixed in its findings. For example, a study of marriage and fertility patterns in 25 sub-Saharan African countries found that exposure to violent events was related to modest reductions in childbearing [24]. However, findings were heterogeneous across settings and subgroups of women and did not focus on fertility in the context of child marriage specifically. Another review of adolescent transitions in the context of armed conflict supports the view that the impact of conflict on adolescent childbearing is mixed, with three studies reporting a decline in fertility and five reporting an increase in childbearing [7]. These dynamics were intimately linked to shifts in incidence and timing of adolescent marriage.

The finding that child brides had longer intervals between marriage and first birth is a positive one and merits further investigation. Qualitative research can help unpack and elucidate the decision-making processes that lead to delayed first birth among child brides. By better unpacking these processes, interventions can support and sustain these processes to ensure that girls married as children are protected from the harmful effects of early childbearing and high fertility. That said, since overall child brides still had lower ages of first birth, interventions to promote delaying childbearing until later ages are needed in this setting.

This study should be considered in light of some limitations. First, our estimates rely on cross-sectional household surveys with girls who were asked to self-report their age and age of marriage, and are thus subject to several biases, including selection and recall bias. Recall bias may have affected the accuracy of data collected on year of birth and reported age. Selection bias may have resulted from the sampling strategy not being based on sampling frames in some locations in Hadramout where a listing of the IDP population was not available. That said, we relied on trusted guides who are deeply embedded in their communities and were thus able to provide detailed insights about the presence and number of IDP families in their neighborhoods. Second, the study only included females, and did not include males. While child marriage disproportionately affects girls, there is some evidence of child marriage occurring among the male population in this context [9]. In addition, males are critical to decisions related to both marriage and childbearing in Yemen and future research should prioritize male engagement to better understand male perspectives on family formation. However, due to logistical constraints, the study did not examine this group and focused instead on females. Because of this tradeoff, we were able to include a large sample of female respondents, which is a strength of the study.

## Conclusion

The study brings to sharp focus the impact of displacement on family formation dynamics, and specifically the risk of child marriage. Those who were internally displaced were significantly more likely to enter into marriage earlier compared to their host counterparts. We also find evidence that child marriage was not immediately followed by childbearing in this context, regardless of displacement status. That child marriage is more prevalent among IDPs but isn’t immediately followed by childbearing underscores the need for interventions that focus on child marriage prevention and those that ensure that delays in childbearing are sustained.

## Data Availability

The dataset supporting the conclusions of this article is available on request.

## Abbreviations

IDPs: Internally displaced persons
JHSPH: Johns Hopkins University School of Public Health
MICS: Multiple Indicator Cluster Survey
